# The economic and societal impact of periodontal and peri‐implant diseases

**DOI:** 10.1111/prd.12568

**Published:** 2024-05-01

**Authors:** Muhammad H. A. Saleh, Debora R. Dias, Purnima Kumar

**Affiliations:** ^1^ Department of Periodontics and Oral Medicine University of Michigan School of Dentistry Ann Arbor Michigan USA; ^2^ Department of Dentistry State University of Maringá Maringá Paraná Brazil

**Keywords:** diagnosis, economic burden, non‐surgical therapy, peri‐implant diseases, periodontitis, prevention, regeneration, resective surgery, societal burden

## Abstract

Periodontal and peri‐implant diseases result from a chronic inflammatory response to dysbiotic microbial communities and are characterized by inflammation in the soft tissue and the ensuing progressive destruction of supporting bone, resulting in tooth or implant loss. These diseases' high prevalence, multifactorial etiology, extensive treatment costs, and significant detriment to patients' quality‐of‐life underscore their status as a critical public health burden. This review delineates the economic and sociocultural ramifications of periodontal and peri‐implant diseases on patient welfare and healthcare economics. We delve into the implications of diagnosis, treatment, supportive care, and managing destructive tissue consequences, contrasting these aspects with healthy patients.

## 1 BACKGROUND

**FIGURE 1:**
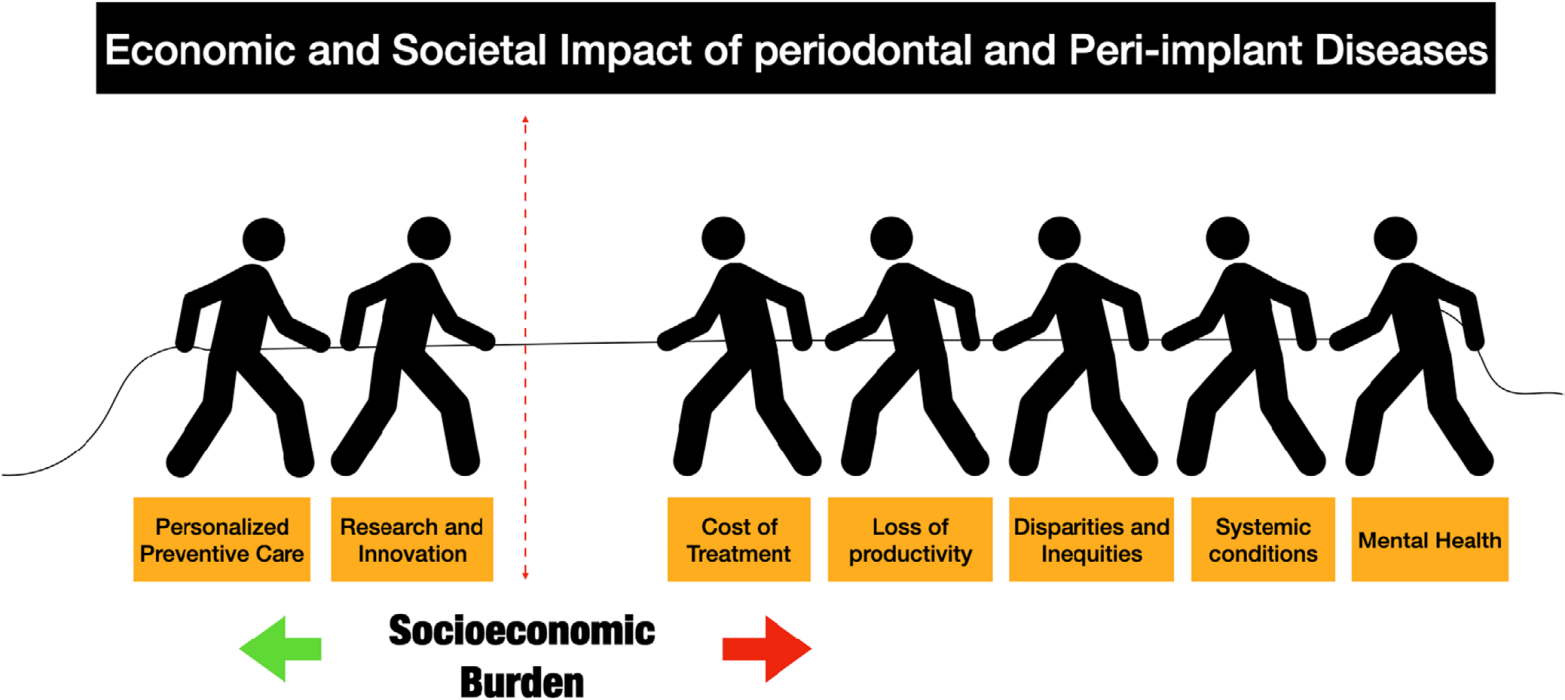
Socioeconomic burden of periodontal and peri‐implant diseases.

Periodontitis is a chronic multifactorial inflammatory disease associated with a dysbiotic microbiome characterized by progressive tooth‐supporting apparatus destruction.^1^ It is characterized by clinical attachment loss and radiographically evidenced alveolar bone loss, the presence of periodontal pocketing, and gingival bleeding.^1^ If untreated, it may lead to tooth loss, although, in most cases, it is preventable and treatable. It is rapidly burgeoning into a major public health problem due to its high prevalence, consequent edentulism, masticatory dysfunction, negative impact on general health, and high dental care costs, especially with increasing lifespans (Figure 1).^1^, ^2^, ^3^, ^4^

Similar to periodontitis, peri‐implantitis is defined as a plaque‐associated pathological disease that affects the tissues that surround and support dental implants. It is characterized by inflammation in the peri‐implant mucosa and subsequent progressive loss of supporting bone.^5^ Clinically, peri‐implantitis sites exhibit inflammation, bleeding on probing and/or suppuration, increased probing depths and recession of the mucosal margin, and radiographic bone loss compared with previous examinations.^5^ As with periodontitis, the disease represents a growing public health problem due to its high prevalence. However, compared to periodontitis, its progression can be accelerated and follow a nonlinear path,^6^ and treatment approaches are not as predictable as around teeth. In addition, treatment of peri‐implant disease accounts for substantial dental care costs, and implant loss creates a negative impact on the patient's quality of life.^7^

Understanding the economic and social impact of these diseases can help create specific actions for the public, policymakers, educators, and professional organizations in prevention, diagnosis, and care.^3^ Therefore, the present review aimed to summarize, in a narrative manner, the impact of periodontal and peri‐implant diseases on the patient's financial perspective and quality of life. The costs and social implications were considered from the perspectives of diagnosis, different treatment approaches, supportive care, and management of sequels of the diseases, and further compared to healthy patients.

An electronic and manual search was conducted in MEDLINE (PubMed database) for relevant articles (i.e., observational studies, randomized/controlled clinical studies, systematic reviews, meta‐analyses, and consensus reports) to answer the following questions: (i) “What is the impact of periodontal and peri‐implant diseases on the costs of health care?” and (ii) “What is the impact of periodontal and peri‐implant diseases on the patient's quality of life?” Data from identified and relevant publications were extracted, and overall findings were summarized in a narrative manner.

## 2 GLOBAL BURDEN OF PERIODONTAL AND PERI‐IMPLANT DISEASES

Periodontal diseases (PD), including gingivitis and periodontitis, are preventable, do not pose significant diagnostic challenges, and can be effectively treated; yet, periodontitis is the major cause of tooth loss in adults worldwide.^3^, ^8^ and is the sixth‐most prevalent disease.^9^, ^10^, ^11^, ^12^ It is estimated that one of two adults have periodontitis.^13^ The recent Global Burden of Disease Study (GBD, 1990–2019) has shown that in 2019, the global burden of severe periodontitis affected around 1.1 billion people.^8^, ^14^ The highest number of prevalent cases of severe periodontitis worldwide was observed in individuals among 40‐ to 49‐year‐old, with an increase in the prevalence rate from 15–19 to 50–54 years, and followed by a gradual decrease among older individuals.^8^ These conditions not only lead to tooth loss, edentulism, and masticatory dysfunction but also impose substantial socio‐economic impacts and healthcare costs.^12^, ^15^

The economic burden of periodontitis extends well beyond the oral cavity; as a chronic noncommunicable disease (NCD), periodontitis shares features of other NCDs like cardiac diseases, diabetes, cancer, and chronic respiratory disease.^16^, ^17^ Bacteria and bacterial products from oral biofilms, as well as mediators from the inflamed periodontal tissues, can disseminate through the body, exacerbating systemic diseases like diabetes, atherosclerosis, rheumatoid arthritis, and pulmonary infections.^18^, ^19^ It is established that periodontitis has a bidirectional relationship with overall health, that is, improvements in periodontal health offer multiple benefits to systemic health and well‐being.^3^, ^8^, ^20^, ^21^ Therefore, the primary prevention of the disease has the potential to reduce the economic and social impact of these systemic diseases.^22^

Active periodontal treatment, when followed by supportive periodontal care, successfully reduces tooth loss^23^, ^24^, ^25^ and improves patients' quality of life.^26^, ^27^ Optimal periodontal health should be accomplished via minimally invasive, cost‐effective methods, acknowledging the patient's enduring dedication to routine maintenance visits to preserve the successful therapeutic outcome.^28^, ^29^ Despite the preventable nature of periodontitis, some patients may not seek treatment due to the symptomless early stages of the disease and low awareness of periodontal health.^30^, ^31^ Additionally, socio‐economic and cultural barriers can prevent the public from accessing preventive approaches, early diagnosis, and treatment.^31^, ^32^ Therefore, an economic evaluation of various periodontal treatment modalities to ascertain which intervention provides the most significant “value for money” is of major public interest.^33^

Peri‐implant diseases have not yet been considered in the Global Burden of Disease Studies but affect about one of five individuals rehabilitated with implants.^34^, ^35^, ^36^ Mirroring periodontitis, the primary etiological factor for the onset and progression of peri‐implantitis, is biofilm accumulation. However, compared to periodontitis, its progression can be accelerated and follow a nonlinear path,^6^ and treatment approaches are not as predictable as around teeth. In addition, peri‐implantitis accounts for substantial dental care costs, and the loss of an implant negatively impacts a patient's quality of life.^7^ Several risk factors/indicators have been identified in previous studies, including a history of periodontitis, inadequate plaque control, and lack of regular supportive care following implant therapy, smoking, diabetes, or local factors such as absence of peri‐implant keratinized mucosa, presence of submucosal cement, or positioning of implants limiting access to oral hygiene and maintenance.^37^, ^38^ The disease progresses rapidly following onset, resulting in loss of the implant as soon as 2 months following diagnosis. Moreover, the predictability of the available treatment approaches in the long term may be uncertain.^7^, ^38^, ^39^, ^40^, ^41^ Therefore, preventive measures for peri‐implantitis have been emphasized as the primary objective for several years. These measures aim to mitigate patient‐level and implant‐level risk factor(s) even prior to implant placement (primordial prevention) or prior to disease onset (primary prevention); and include regular supportive care, keratinized mucosa augmentation if necessary, smoking cessation, and promotion of healthy behaviors.^38^ To fully investigate the efficacy of primordial and primary preventive methods in decreasing disease incidence or recurrence, it is crucial to first delineate the burden of these diseases.

Understanding these diseases' economic and social impact will enable specific actions for the public, policymakers, educators, and healthcare providers focused on prevention, diagnosis, and care.^3^ Therefore, the present review aims to summarize, narratively, the impact of periodontal and peri‐implant diseases on the patient's economic aspect and quality of life. The costs and social implications were considered from the diagnosis, different treatment approaches, supportive care, and management of sequels of the diseases, and further compared to healthy patients.

An electronic and manual search was conducted in MEDLINE (PubMed database) for relevant articles (i.e., observational studies, randomized/controlled clinical studies, systematic reviews, meta‐analyses, and consensus reports) to answer the following questions: (i) “What is the economic impact of periodontal and peri‐implant diseases and cost‐effectiveness of the provided therapy?” and (ii) “What is the impact of periodontal and peri‐implant diseases on the patients quality of life?”. Data from identified and relevant publications were extracted, and overall findings were summarized narratively.

## 3 THE VICIOUS CYCLE OF PERSONAL SOCIOECONOMIC STATUS AND THE GLOBAL DISEASE BURDEN

Socioeconomic status has been increasingly recognized, as a crucial determinant of oral health, not merely as a confounding variable for the identification of risk indicators for periodontitis.^42^, ^43^, ^44^ A meta‐analysis by Boillot et al.^45^ found that lower socioeconomic status was associated with adverse periodontal conditions. This relationship remained significant even after controlling for other factors. Other multiple reviews addressed this topic. Some critical and systematic reviews studied both cross‐sectional and longitudinal data,^46^, ^47^ while Schuch and co‐workers focused solely on longitudinal data.^48^ Whether it was performed on a particular demographic region,^49^ a particular age group,^50^ or individuals away from their homeland,^51^ each of these reviews concluded that lower socioeconomic status correlated with poor periodontal health.

A more recent review highlighted a strong association between low socioeconomic status and an increased prevalence of periodontitis, even after controlling for behavioral variables such as smoking.^52^ Certain behaviors influenced by socioeconomic adversity, for example, inadequate oral hygiene or inconsistent dental visits, contributed to the prevalence of periodontitis in economically disadvantaged groups. However, other factors are also at play, as demonstrated by several studies that continued to identify a significant relationship between socioeconomic status and periodontitis after adjusting for these behaviors.^53^, ^54^ A plausible biological explanation for this association is the concept of allostatic load, which encapsulates the chronic effects of stress on the body's biological systems.^52^ Chronic stress, created by low socio‐economic status and its associated lifestyle challenges, induces structural and functional changes in the biological systems responsible for maintaining stability or allostasis. This persistent alteration in neurological, neuroendocrine, and immune systems elevates the risk of various diseases^55^ since chronic stress can exhaust the body's adaptive capabilities, creating an imbalance in regulatory molecules.^56^ This long‐term exposure to stress can accelerate aging via physiological dysregulation, thereby influencing disease occurrence.^56^

**FIGURE 2:**
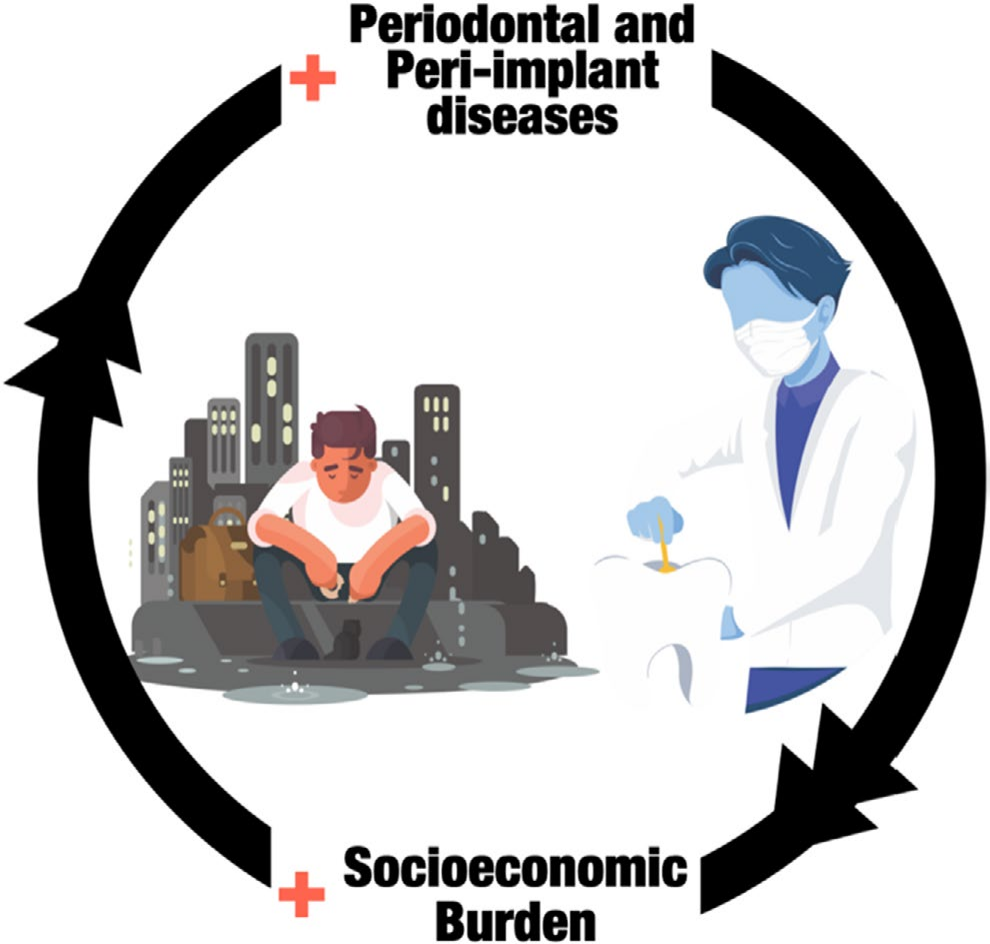
The two‐way relationship between periodontal and peri‐implant diseases and its socioeconomic impact.

Epidemiological studies indicate a correlation between periodontitis and various systemic conditions, including ischemic atherosclerotic disease, obesity, metabolic syndrome, hypertension, adverse pregnancy outcomes, Alzheimer's disease, and preeclampsia. Various theories have been suggested to elucidate the impact of periodontitis on these systemic conditions.^57^ It was suggested that allostatic markers, systemic inflammation, and socioeconomic and behavioral factors collectively contribute to the development of periodontitis and other systemic disease.^58^ It was observed that the correlation between socioeconomic factors and periodontitis and ischemic heart disease was reduced after accounting for allostatic load.^58^ Another study reported that the odds ratio between most systemic inflammatory markers and periodontal disease reduced after adjusting for socioeconomic status.^59^ These findings serve to highlight the complex interrelation that exists among socioeconomic status, allostatic load, and systemic inflammation (Figure 2).

## 4 THE ECONOMIC BURDEN OF PERIODONTAL AND PERI‐IMPLANT DISEASES

### 4.1 Calculating the burden

Calculating oral disease statistics has often posed a challenge due to suboptimal examination conditions, varying detection methods, evolving case definitions, and resource constraints.^60^ While some countries have initiated national surveys or surveillance systems to document oral health status, most have not. Efforts to quantify global dental disease prevalence have been made, but determining the global burden of oral diseases has been elusive until recently.^11^, ^61^

In 2013, a significant shift occurred when Marcenes and colleagues^11^ published the first paper estimating the global oral disease burden using data from the Global Burden of Disease (GBD) study. This provided the first plausible estimate of the global oral disease burden. The GBD study group has since published updated estimates, including data up to 2015.^9^ In 2017, Kassebaum et al. offered a piece of updated information on global oral health issues, aligning with the latest GBD 2015 estimates.

In periodontology, similar to all healthcare fields, economic considerations are crucial due to the scarcity of governing resources, time, and finances. Decisions regarding the best use of resources must be made, and healthcare interventions must be evaluated accordingly.^33^, ^61^ The assessment of interventions can be classified into four categories: efficacy, effectiveness, availability, and efficiency.^62^ Efficiency evaluations can illuminate better ways to allocate resources within periodontal care or through other health programs.^62^, ^63^

### 4.2 Cost analysis

Typically, economic evaluations compare inputs with outcomes or costs with benefits. Multiple types of economic evaluations may be used^62^, ^64^:

1. Cost Minimization Analysis (CMA): This is used when the outcomes of two or more interventions are assumed to be equivalent. The analysis focuses solely on comparing costs; the cheapest option is considered the most efficient. Cost minimization analysis can provide helpful information that is of little use in decision‐making.
2. Cost‐Effectiveness Analysis (CEA): This compares an intervention's costs and health effects to assess whether it is a good value for money. It measures benefits in natural units such as disease‐free days. The results are presented as a ratio of cost per unit of health outcome, for example, cost per life year gained. However, these measures often overlook the patient's broader health impact, calling for health‐related quality‐of‐life measures.
3. Cost–Utility Analysis (CUA): This is a form of CEA that considers the quality‐of‐life years (QALYs) and disability‐adjusted life years (DALYs) gained by the intervention. It allows for the comparison of different types of health outcomes.
4. Cost–Benefit Analysis (CBA): This compares the costs and benefits of an intervention, with both measured in monetary terms. The aim is to see if the benefits outweigh the costs.
5. Cost–Consequence Analysis (CCA): This lists all the costs and outcomes of the interventions separately and provides a detailed picture of the costs and outcomes to allow a decision to be made.
6. Budget–Impact Analysis (BIA): This estimates the financial consequences of adopting a new intervention. Healthcare decision‐makers often use this method.
7. Cost‐of‐Illness (COI) Studies: These aim to quantify the total economic burden of a specific disease or health condition. This can include direct costs (like healthcare expenditures), indirect costs (like lost productivity), and intangible costs (like pain). Cost minimization disregards effectiveness and is most applicable when comparing equally effective treatments. Although it provides valuable cost information, without measures of effectiveness, it has limited utility in decision‐making.

The three‐most common analyses used in the field of periodontics implant dentistry are cost minimization analysis (CMA)^65^, ^66^ and cost‐effectiveness analysis (CEA).^67^, ^68^ Limited work has been done in oral health using utilities or contingent valuation, and even fewer applications of these findings in cost–utility or cost–benefit analyses.^69^ Measures like the quality‐adjusted tooth year (QATY) have been developed but have seen limited success.^70^

**FIGURE 3:**
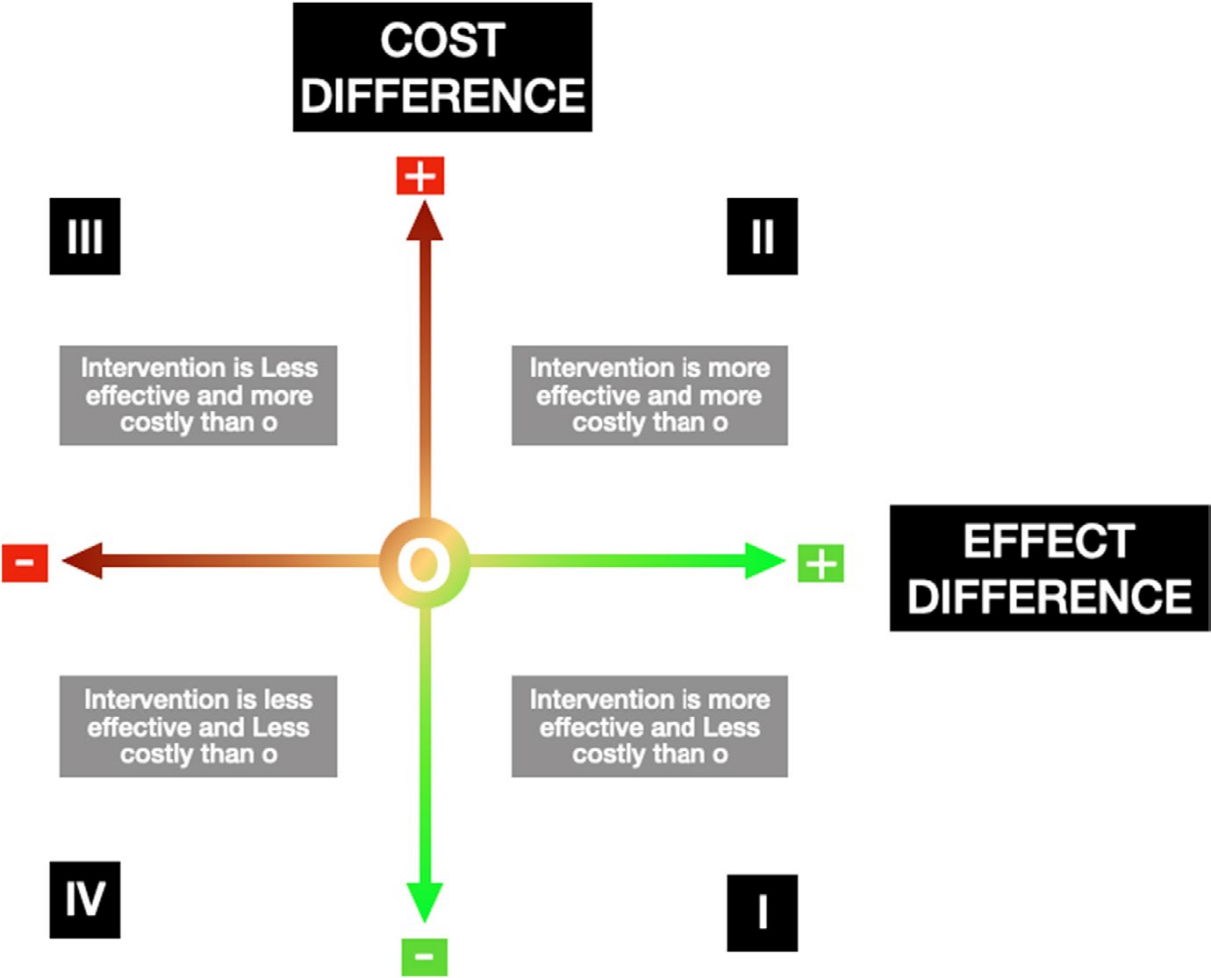
In Quadrants I or III, the choice between the programs is clear. In Quadrant I, the intervention of interest is both more effective and less costly than the alternative. That is, it dominates the alternative. In Quadrant III, the opposite is true. In Quadrants I and III, the choice depends on the maximum cost‐effectiveness ratio one is willing to accept. The choice between II and IV is a function of the magnitude and clinical relevance of the effect, as well as the economic burden on patients. Adapted from Black, W.C. The cost‐effectiveness plane: a graphic representation of cost‐effectiveness. Medical Decision Making, Volume 10, Number 3, pp. 212–15, Copyright © 1990 by Society for Medical Decision Making. Reprinted by permission of SAGE Publications.

Multiple literature reviews evaluated the types^33^, ^68^, ^71^, ^72^ and quality^72^ of economic evaluations performed during the clinical management of periodontal diseases. In periodontics, CEA may be used to determine whether one treatment might result in fewer teeth lost but is also costlier. The incremental cost‐effectiveness ratio (ICER) is used to evaluate cost‐effectiveness. A lower ICER signifies a more cost‐effective method. In a CUA, the costs of the two treatments and their effects on the patient's quality of life, measured QALYs, will be assessed. This might use an Oral Health Impact Profile (OHIP). In a CBA, the costs of the two treatments with the monetary benefits of preventing tooth loss. This might involve estimating the cost savings from avoiding the need to replace lost teeth, the increased productivity from maintaining good oral health, and even the increased happiness and self‐esteem from maintaining a healthy smile (Figure 3).

### 4.3 Economic analyses for periodontal diseases

**FIGURE 4:**
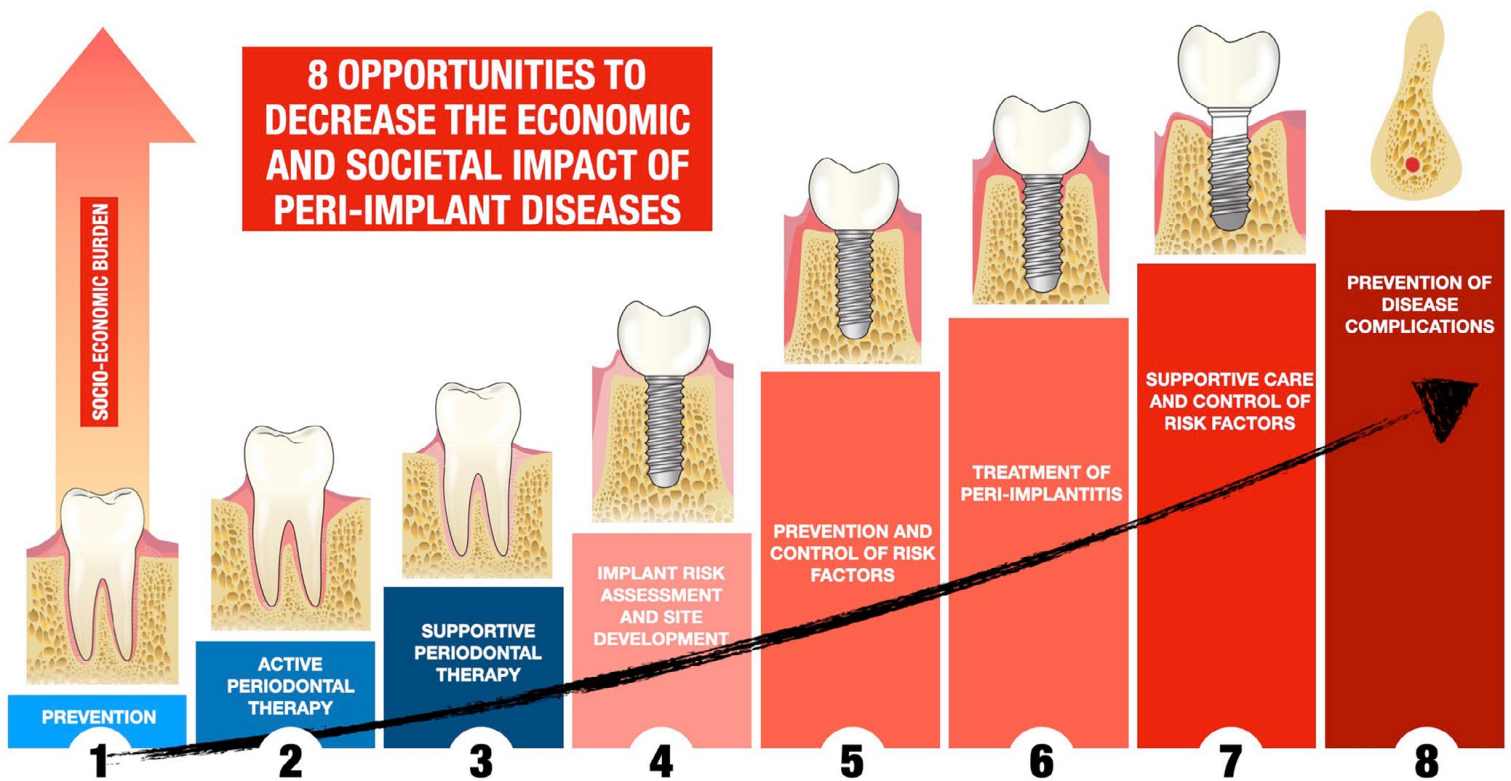
Eight opportunities to decrease the economic and societal impact of peri‐implant diseases: (1) Primary prevention, including management of risk factors and promoting healthy behaviors; (2) Active periodontal therapy; (3) Supportive periodontal therapy, the secondary prevention of the disease; (4) Implant risk assessment and site development, a primordial prevention approach that focuses on individuals who do not have yet received dental implants, avoiding risk factor exposure; (5) Prevention and control of risk factors, that is, primary prevention in individuals with healthy tissues but exposed to risk factors; (6) Treatment of peri‐implantitis; (7) Supportive care and control of risk factors to prevent disease recurrence, the secondary prevention of the disease; (8) Tertiary prevention, that is, prevention of disease complications by delaying the progression of the disease to avoid implant loss.

Prevention, early diagnosis, and targeted intervention provide a multi‐hit approach to decrease the economic and societal impact of peri‐implant diseases (Figure 4). The following sections will discuss the evidence that supports the efficacy of each of these intervention opportunities.

#### 4.3.1 Personalized preventive measures and early diagnosis

Periodontitis can be prevented through effective management of gingivitis and the promotion of healthy lifestyles at the population and individual levels.^22^ It can be executed through professional instruction of self‐performed oral hygiene. A second tactic guided by the common risk factor approach is a comprehensive, population‐level approach to health education.^73^ It is crucial to individualize prevention by diagnosing and assessing risk profiles. This ensures that each person's unique needs are addressed. The collective consensuses from several research workshops emphasized the need for comprehensive oral healthcare and its role in overall health.^2^, ^4^, ^18^, ^74^, ^75^ Diseases like periodontitis necessitate a holistic approach to prevention and treatment.^7^ Therefore, the WHO's common risk factor approach should include self‐performed oral hygiene as an integral lifestyle factor. National preventive programs must recognize oral health as a fundamental part of general health, incorporating it into large‐scale health efforts when possible.^3^, ^76^

Periodontitis can be diagnosed through comprehensive periodontal evaluation, a routine part of dental visits. While affordable and sensitive screening methods have been introduced, their universal adoption remains challenging, leading to a high burden of undiagnosed diseases worldwide.^3^ Delayed diagnosis often increases disease management costs and can be a significant source of litigation.^77^ Early diagnosis, followed by suitable treatment and secondary prevention, can improve quality of life, preserve dental function, and decrease the financial burden of periodontitis management.^3^, ^22^, ^62^, ^77^ Early diagnosis, followed by suitable treatment and secondary prevention, can improve quality of life, preserve dental function, and reduce the financial burden associated with disease management.^3^, ^22^

Diagnosis must include an assessment of risk factors, including systemic conditions. Since individuals in developed countries often have more frequent dental visits than medical check‐ups, oral healthcare professionals can play a significant role in detecting undiagnosed conditions like diabetes, hypertension, and obesity.^78^ This expanded role can enhance patients' overall health, strengthen the healthcare team, and decrease the financial burden on the patient.^78^

Multiple studies performed economic evaluations for early preventive phases of periodontal therapy preventative programs and diagnosis.

##### Prevention

In a cost‐effectiveness analysis (CEA), Jönsson et al.^79^ compared an individually tailored oral health educational program (ITOHEP) based on the cognitive behavioral strategies integrated into nonsurgical periodontal treatment. They compared it with a standard treatment program. This CEA concluded that a personalized OHE program is more cost‐effective and results in higher treatment success rates than a standardized one.

A landmark cost–benefit RCT which recruited 1877 participants tested the clinical effectiveness and assessed the economic impact of personalized OHA (oral health advice) versus routine OHA, 12‐monthly prophylaxis (scaling and polishing) compared with 6‐monthly prophylaxis, and no prophylaxis compared with 6‐monthly prophylaxis.^80^ Personalized OHA with 6‐monthly prophylaxis had the most significant incremental net benefit (£48). However, no difference in the therapeutic endpoints was found between all groups.^80^

##### Genetic testing

Higashi et al.^81^ evaluated the economic impact of genetic testing compared to no genetic testing.^9^ Eight sub‐cohorts were followed using a Markov model^82^ to assess the varying cost and quality‐adjusted life‐years (QALYs) accumulated over 30 years. Through the application of varied modeling approaches over 30 years, the genetic testing yielded outcomes that spanned from a cost reduction of $830 140 with 52.8 fewer instances of severe periodontitis to an increased expenditure of $300 430 and an additional 3.6 severe periodontitis cases per 1000 patients. Three critical factors influenced the results of that study: (1) the adherence rate to maintenance therapy in test‐positive as opposed to nontested patients; (2) the efficacy of nonsurgical intervention; and (3) the comparative risk of disease progression in test‐positive patients.^81^ The authors highlighted the lack of differentiated prevention and treatment methods for patients with high and low risk of periodontal disease. They noted that without these distinct approaches, the cost–benefit of identifying susceptibility to the disease is unlikely to be substantial. Again, this implies the need for tailored strategies based on patients' risk levels to maximize the effectiveness of periodontal disease management.

#### 4.3.2 Treatment

Periodontal therapy aims to manage gingivitis and periodontitis, halt disease progression to prevent tooth loss, maintain a functional dentition for life, and enhance the quality of life and self‐esteem.^3^ Longitudinal studies revealed that after active periodontal therapy (APT), tooth loss averages 0.1 tooth/patient/year in subjects participating in supportive periodontal care (SPC) in specialist practices.^83^ This rate should be consistent with most subjects' lifetime preservation of dentition. A higher tooth loss rate is expected for older individuals and smokers.^84^, ^85^

Delivery of periodontal treatment necessitates a highly skilled, sometimes highly specialized, oral healthcare professional. However, accessing such specialists can be a challenge in numerous healthcare systems. Luckily, periodontal therapy comprises a series of sequential phases that increase in complexity and dependent on each other. Reciprocally, the training and skill level of the oral healthcare professional expected to accomplish these phases increase. These phases include risk factor control and promotion of healthy lifestyles; surgical correction of anatomical lesions for patients with persistent periodontal pockets; rehabilitation of masticatory function and aesthetics in advanced cases; and stringent monitoring and high‐level care for patients at higher risk of recurrence.^86^, ^87^

Considering the global burden of disease, proper periodontal care involves a highly trained and motivated oral health professional team.^3^ As treatment complexity increases with disease progression, planning suitable primary and secondary care in national health systems is crucial. Primary care is usually provided in general dental offices through dentist‐led teamwork with dental hygienists and assistants in countries with structured dental services. Secondary care is delivered in specialized centers under a specialist's guidance. These centers manage more advanced periodontitis cases and those with complex medical comorbidities.^3^

The successful management of periodontitis involves raising public and professional awareness about early signs, improving access to care, informing about the standard of care for different disease stages, and addressing misconceptions about self‐care or self‐medication.^17^ It is also essential to highlight the impacts of incomplete or ineffective treatment, the role of dental implants in rehabilitating masticatory dysfunction, and the significant progress made in periodontal treatment over the last decades.^1^, ^88^ Addressing these priorities and availing the necessary resources can improve access to care and contribute to managing the current disease burden.

##### Nonsurgical versus surgical therapy

A study analyzing cost utility in periodontal treatment methods used an expert panel of periodontists and a patient survey to establish the quality‐adjusted tooth years (QATY) for five treatment strategies, ranging from semiannual prophylaxis to osseous surgery.^89^ The study concluded that nonsurgical methods were more cost‐effective than surgical ones, with antibiotic use yielding the lowest cost‐effective ratios. However, this study's limitation was its reliance on expert opinion to predict outcomes like tooth life expectancy. In a randomized controlled trial,^90^ immediate open‐flap debridement cost minimization was compared against nonsurgical scaling and root planing. The two treatment groups achieved comparable clinical outcomes after 3 years, with the nonsurgical approach saving €654 per patient. However, more patients in the nonsurgical group required additional scaling and root planing during supportive care.

In a CUA questionnaire aimed at understanding the willingness to pay for monthly dental insurance premiums, various treatment options were evaluated against each other. These options included nonsurgical therapy, surgical treatment, full‐mouth extractions, and no treatment at all. The study revealed that individuals, regardless of age or potential risk for periodontal disease, expressed readiness to pay extra insurance premiums for their preferred treatment method. Interestingly, the majority (71%) of participants, cutting across all income brackets, leaned toward periodontal surgery for moderate‐to‐advanced periodontal disease. The main motivation behind this preference was the lower risk of tooth loss associated with surgical intervention.^91^

##### Local delivery systems

A systematic review performed a cost‐effectiveness analysis to compare locally delivered tetracycline fibers, chlorhexidine chips, minocycline microspheres, and doxycycline gel. The results demonstrated that for single teeth, tetracycline delivery was the most cost‐effective approach, while for quadrants, tetracycline and chlorhexidine delivery had similar cost‐effectiveness.^92^ Another study with a similar methodology observed that local delivery of minocycline was cost‐effective if patients' WTP threshold exceeded £1800 for a 1 mm attachment gain on all affected teeth. However, this study also did not conduct sensitivity analyses.^69^

##### Bioactive agents (Biologics)

The utilization of bioactive products “biologics” in periodontal therapy was also investigated. Using enamel matrix derivatives combined with bioactive glass or bovine bone substitutes emerged as the most cost‐effective if the willingness to pay (WTP) was at least €150–175 per incremental millimeter of probing depth reduction or CAL gain.^93^

##### Resective versus regenerative therapy

Regenerative approaches for the treatment of intrabony defects frequently result in improved outcomes and longer tooth retention. However, the regenerative procedure is frequently more expensive than the access flap alone. Cortellini et al.^94^ compared the long‐term outcomes and costs of three treatment approaches for intrabony defects. Sites randomly received modified papilla preservation flaps with titanium‐reinforced expanded polytetrafluoroethylene (ePTFE) membranes (1183€), access flaps with expanded e‐PTFE membranes, and access flap alone (549€). After 20 years under regular supportive care, sites treated with flap alone were the ones with an incidence of tooth loss and higher attachment loss. At the long‐term follow‐up, sites treated with flap procedures had higher chances of disease recurrence and higher costs of re‐interventions than regenerated sites (501.27 ± 210.54 € vs. 159.00 ± 88.95 €, respectively). Considering the costs of supportive periodontal care, the total costs of treatment at 20 years were 3090.98 ± 210.66 € for flap alone, 3382 ± 88.95 € for flap and e‐PTFE, and 3322.79 ± 54.14 € for MPPT and e‐PTFE groups. Therefore, periodontal regeneration procedures may require a higher initial cost, but it result in higher tooth retention and arrest its progression. An RCT^95^ compared periodontal regeneration or tooth extraction and implant or prosthetic replacement of teeth with attachment loss to the apex. Fifty Stage III or IV periodontitis patients were treated, and after 10 years, they were re‐evaluated. Recurrence analysis revealed that the 95% confidence interval of the costs was significantly lower for periodontal regeneration than for tooth extraction and replacement. Patient‐reported outcomes and OHRQoL were improved in the two groups. The authors concluded that “periodontal regeneration can be considered a clinically viable and more economical alternative to extraction and replacement for teeth severely compromised by the presence of an intra‐bony defect involving the apical portion of the root.”

##### Surgical therapy versus extraction and replacement

Cortellini et al.^96^ investigated the cost‐effectiveness of periodontal regeneration versus extraction and tooth replacement with an implant or a fixed partial denture. During a 10‐year follow‐up, the average cumulative costs in euros associated with managing recurrences for regenerated teeth and prosthetic tooth replacement were analyzed. Periodontal regeneration yielded significant gains in clinical attachment levels, with an 88% tooth survival rate over 10 years. The survival rates, as well as complication‐free survival metrics, remained similar for both the regenerated teeth and those subjected to extraction and replacement. From an economic perspective, periodontal regeneration proved to be more cost‐efficient, with almost half of the costs incurred. Both groups exhibited enduring enhancements in patient‐reported outcomes and quality of life.^96^

Simulations based on the German health system have indicated that tooth retention after complex periodontal therapy of teeth with furcation involvement is more cost‐effective than their extraction and replacement with an implant‐supported fixed partial denture.^97^ Another study assessing the actual cost of retaining molars in the same health system showed the minimal costs for retaining periodontally compromised molars.^98^

##### Specialist‐delivered supportive periodontal care

Secondary prevention, that is, supportive care, is pivotal to avoiding disease recurrence. Schwendicke^99^ compared the cost‐effectiveness of regular versus irregular supportive care using a tooth‐level Markov model, which included the replacement of 50% of removed teeth using implant‐supported reconstructions. The authors observed that regular SPC was more effective (28.7 vs. 26.1 years of tooth retention) but more costly (806 vs. 731 €per tooth) than irregular care. The incremental cost‐effectiveness ratio (additional costs per tooth retention year) of regular SPC was 29 €/year. Similarly, Fardal and Grytten,^68^ in a study performed in a private practice setting in Norway, have shown that regular maintenance care was associated with reduced tooth loss than irregular maintenance, considering follow‐ups of 16–26 years; additionally, the yearly cost of maintaining a tooth was estimated to be 20.2 €.

##### Routine care versus specialist periodontal programs

Does SPC need to be done in a specialist clinic? This tends to be a common question in daily practice. In a study by a Malaysian group examining the cost‐effectiveness of periodontal disease treatment, the incremental cost‐effective ratios were found to be MYR 451 (99$ in July of 2023) per additional quality‐adjusted life year (QALY) and MYR 5713 (1255$ in July of 2023) per additional mm clinical attachment level (CAL) gained.^100^ It was thus more cost‐effective for the public sector to provide specialist periodontal treatment only for patients with moderate and severe periodontitis.^100^ A very similar result was reported by Martin in 2014, who reported that in patients with low risk and with mild chronic periodontitis, the cost of periodontal treatment to preserve one tooth is higher than a single tooth replacement and a three‐unit fixed bridge. Thus, periodontal treatment could be justified on the exclusive basis of tooth preservation when the prognostic risk is moderate or high,^101^ regardless of the current disease severity.^66^, ^102^

##### General considerations in an aging population

People with chronic conditions, such as diabetes and cardiovascular diseases, who are often older adults, are at higher risk for oral conditions.^103^ Periodontitis and associated tooth loss are also prevalent in older adults and are associated with a plethora of chronic diseases.^104^ This interplay between oral health and systemic health may create a vicious cycle of deteriorating health and lead to increased severity of both periodontitis and chronic conditions, escalating the cost of management of both.

In the USA, the population aged 65 and above is increasing. Currently, 54.1 million Americans, or 1 in 6, are over 65 years old.^105^ By 2030, this will rise to 1 in 5, or about 70 million people, and by 2060, it will reach 98.2 million.^106^ This older population is diverse in terms of race/ethnicity, socioeconomic status, health, and functional status.^107^ Around 80% of older Americans have at least one chronic disease, and nearly 70% have at least two.^108^ As these diseases progress, physical and neurobiological changes can lead to disability. The CDC reports that 40% of people older than 65 have a physical or cognitive disability,^109^ which can impact oral self‐care and access to oral healthcare.

Some priorities have major implications for policymakers as health systems need to adapt to the challenge of system‐wide changes to enable (promote) tooth retention later in life and management of deteriorating oral health in increasingly dependent elders.^4^ With an aging population and higher expectations for oral health, more older Americans are seeking dental care. Despite advancements in the prevention and treatment of oral diseases, older adults are still at risk for many oral diseases, including periodontitis.^4^ Despite the desire to maintain natural teeth, societal attitudes and healthcare policies often hinder oral healthcare for older adults. With nearly 1 in 10 Americans aged 65 years and older experiencing severe periodontitis.^110^, ^111^ General dentists who encounter patients with periodontitis may refer these patients to see a periodontist for specialty care.^110^ It is a significant public health problem that needs to be addressed. As the US population ages and more older adults retain more teeth, they become more susceptible to periodontitis and its complications.^110^ The dental profession will be required to provide dental care to people with more complex health histories who may have a reduced capacity to tolerate dental care.^112^

Solowiej‐Wedderburn studied the economic benefit arising from enhanced glycemic regulation through periodontal intervention and stringent maintenance therapy in newly diagnosed patients with Type II diabetes as measured through glycated hemoglobin levels.^113^ The study demonstrated that it was a cost‐effective healthcare strategy. The results were contingent on the percentage of patients adhering to SPC.

### 4.4 Economic analyses in peri‐implantitis

The evolution in the quality of materials, techniques, and proficiency of practitioners has enabled adults to replace their missing teeth more rapidly and successfully than ever before. Contemporary dental implants, whether restored with fixed or removable restorations, demonstrate high durability and esthetic fidelity. The success rates of these implants are among the highest in surgical implant procedures, with a survival rate exceeding 80% for implant‐supported dentures after a quarter‐century.^114^

Due to their enhanced durability, aesthetics, and supportive features, dental implants have emerged as the treatment of choice for many older adults who can afford them.^115^ The prevalence of dental implants has seen a significant upswing, rising from 0.7% during 1999–2000 to 5.7% in 2015–2016.^116^ The most pronounced absolute surge in prevalence, 12.9%, was observed in individuals aged 65–74 years, whereas the most remarkable relative increase, approximately 1000%, was noted among those in the 55‐ to 64‐year‐old bracket. The projected prevalence for 2026 varies from a conservative 5.7% to an upper limit of 23%.^116^

A Swedish study^117^ assessed the costs associated with implant‐supported restorations during the long‐term follow‐up of 514 patients. Over a mean follow‐up of 8 years, on average 12 interventions (preventive and complication‐related procedures) were performed, and costs ranged from 878 € for individuals with single‐tooth restorations to 1210 € for full‐arch restorations. Most of the costs were attributable to preventive measures. Complication‐related costs were 557 € for single restorations and 769 € for full arches. Implant loss was the costliest complication. Thus, costs for supportive care of implant‐supported rehabilitations are associated with the extent of the initial therapy (single tooth vs. full arch).

The cost comparison of supportive care for implants and teeth was compared by Fardal & Grytten.^118^ Among 43 patients with 847 teeth and 119 implants, the mean number of “disease‐free years” was 8.66 for implants, 9.08 for neighboring teeth, and 9.93 for contralateral teeth, with no statistically significant differences. Even though the number of disease‐free years was the same, the extra cost of maintaining implants was five times higher than for teeth due to the high prevalence of peri‐implantitis.^118^

## 5 THE SOCIETAL IMPACT OF PERIODONTAL AND PERI‐IMPLANT DISEASES

Clinical measurements such as probing pocket depth, clinical attachment level, recession, plaque scores, and bleeding on probing are performed every day by clinicians to assess the status of periodontal health or diagnose periodontal diseases. These parameters are fundamental for the clinical and research scenario.^119^, ^120^ However, the impact of the diagnosis and treatment of periodontitis on patients' lives is significantly more than just reducing millimeters of pocket depth. Increasing attention has been placed beyond the clinical outcomes of the treatment, recognizing the impact of the disease process, that is, diagnosis, treatment, and supportive care, on the functional and psychological well‐being of the patients.^121^ This paradigm shift from a treatment‐ to a patient‐centered approach allows for a more holistic standard of care. Numerous questionnaires and scales have been proposed in the literature to evaluate the impact of the disease on patients' quality of life.^119^, ^120^

The World Health Organization defined quality of life as “an individual's perception of their position in life in the context of the culture and value systems in which they live and in relation to their goals, expectations, standards and concerns.”^122^ Oral health‐related quality of life (OHRQoL) is defined as a “multidimensional construct that includes a subjective evaluation of the individual's oral health, functional well‐being, emotional well‐being, expectations and satisfaction with care, and sense of self.”^123^ The OHRQoL can be assessed using questionnaires, scales, and/or related forms to assess the physical, psychological, and social well‐being, with a focus on the possible impact that any oral disease and/or treatment have on different dimensions of a person's life.^119^ Qualitative research, single‐item scales, and composite measures have been used to evaluate the quality of life. To assess subjective perceptions of OHRQoL, the most widely used scales are the visual analog scale (VAS) and the Likert scale.^120^ The oral health impact profile (OHIP), the oral impacts on daily performances (OIDP), the Geriatric/General Oral Health Assessment Index (GOHAI), and the UK oral health‐related quality of life measure (OHQoL‐UK) are the most commonly used questionnaires with composite measures of quality‐of‐life scale (for further information, see Graziani & Tsakos^120^). The OHIP‐14 is a concise tool that has been widely tested and validated in different populations. It contains 14 questions within 7 conceptual domains: functional limitation, physical pain, psychological discomfort, physical disability, psychological disability, social disability, and handicap.^121^, ^124^, ^125^ Responses are based on a 5‐point scale in which participants report whether the impact has affected them never (0), hardly ever (1), occasionally (2), fairly often (3), or very often (4). The OHIP‐14 sum score ranges from 0 to 56, in which the higher the score, the poorer OHRQoL.^126^

### 5.1 Impact of periodontitis on the quality of life

#### 5.1.1 Diagnosis of periodontitis

A plethora of studies has shown that periodontitis has a negative impact on a patient's quality of life.^120^, ^121^, ^127^, ^128^, ^129^, ^130^, ^131^, ^132^ The disease may be “silent” in the early stages since patients often have no or very few symptoms (spontaneous bleeding and swollen gums) years before seeking professional care.^132^ With disease progression, however, symptoms, including sore and receding gums, tooth mobility, pathological migration of teeth, and halitosis, have a major impact on the patient's physical, social, and psychological aspects of life, compromising function, comfort, appearance, and self‐confidence.^133^, ^134^, ^135^ The worst outcome of periodontitis is tooth loss, and in the case of multiple elements, decreased chewing efficacy is also commonly observed, leading to an unhealthy diet with lower nutritional intake.^136^, ^137^ At this stage, these patients often express feelings such as fear of losing the entire dentition, shame of the signs and symptoms of the disease, and anger toward the previous dentist who had not alerted them to a diagnosis of periodontitis.^138^, ^139^

Recently, Wong et al.^121^ assessed, in an umbrella review, the impact of periodontal disease and periodontal treatment on general (HRQoL) and oral health‐related quality of life (OHRQoL). Eight systematic reviews were included: two reported a significant impact of oral conditions on HRQoL,^140^, ^141^ three observed that periodontal disease impairs OHRQoL,^43^, ^132^, ^142^ and three established that periodontal treatment could improve OHRQoL.^143^, ^144^, ^145^ A summary of the available evidence is shown in Table 1.

**TABLE 1:** The societal impact of periodontitis and peri‐implantitis in different scenarios.

Evidence	Periodontitis	Peri‐implantitis
Healthy	Healthy patients have a better OHRQoL^175^	Patients rehabilitated with dental implants are highly satisfied^182^, ^183^ Only 2/3 are aware of the risk for complications^182^
Diagnosis	OHRQoL in patients diagnosed with periodontitis is significantly impaired^127^, ^128^, ^129^, ^130^, ^131^ depending on: Disease severity^135^, ^156^, ^157^, ^158^, ^159^, ^160^, ^161^, ^169^ Stage^160^, ^161^ Grade^160^, ^161^ Extent^163^	OHQoL can be impaired in the presence of peri‐implantitis^184^, ^185^ Most cases are asymptomatic and not perceived by the patients^186^, ^191^
Treatment	Different treatment approaches can improve ORHQoL^125^, ^132^, ^169^, ^170^, ^171^, ^172^, ^173^ and reduce the emotional burden^138^ The quality of life in patients diagnosed with periodontitis may be significantly reduced in patients treated with surgical vs. nonsurgical treatment^172^ Stage IV periodontitis, regenerative approach + orthodontic treatment may improve OHRQoL^215^	The impact on OHRQoL is low before and might stay low up to 3 years after peri‐implantitis surgery^190^
Maintenance	A high OHRQoL can be achieved and maintained long‐term after periodontal treatment,^174^ ^,216–218^ and it may be similar to healthy/gingivitis scores^175^ Supportive care improves OHRQoL Patients without regular care have more tooth loss and poorer OHRQoL^176^	
Sequel management	Following tooth loss, oral rehabilitation improves OHRQoL^181^	Following implant loss, patients may be reluctant to undergo the same procedure in the same clinic/professional^198^

Periodontitis and systemic health have a close relationship. The disease can work both ways with other systemic diseases by different mechanisms, including increasing inflammation levels and its psychosocial impacts. Its interaction with diabetes mellitus, atherosclerosis, and cardiovascular diseases is well known in the literature, as mentioned in the economic section.^18^, ^75^, ^146^, ^147^, ^148^ The two‐way relationship between periodontitis and diabetes mellitus, for example, has a direct impact on a patient's general and oral quality of life since poorly controlled diabetes is considered a risk factor for increased periodontitis severity and, on the opposite way, periodontitis is linked with poor glycemic control in diabetic patients.^149^ Moreover, psychological status, periodontal health, and quality of life are also associated. Heavy workload, social class, lack of sleep, stress, and unhealthy lifestyles may predispose individuals to periodontal diseases.^150^, ^151^, ^152^, ^153^

The severity and extent of the disease play an important role in patients' OHRQoL.^135^, ^154^, ^155^, ^156^, ^157^, ^158^, ^159^ Buset et al.^132^ included 28 studies in a systematic review and observed a significant association between periodontal diseases and OHRQoL. A dose–response type of relationship was identified in eight studies, in which the greater the disease severity or extent, the worse OHRQoL. These findings are in agreement with recent studies that accessed the association of stage and grade of periodontitis with poor quality of life.^160^, ^161^, ^162^ Undeniably, localized forms of the disease also do not impact the quality of life as much as generalized forms.^163^

#### 5.1.2 Treatment of periodontitis

Periodontitis is a treatable and controllable disease. Numerous studies have shown that periodontal therapy can improve the patient's quality of life.^125^, ^130^, ^164^, ^165^, ^166^, ^167^, ^168^, ^169^, ^170^, ^171^ In general health, it is expected that the treatment of the disease will reduce the systemic inflammatory burden. Orlandi et al.^165^ investigated, in a systematic review, the effects of the treatment of periodontitis on systemic health and quality of life. The authors observed that treatment of periodontitis resulted in improved systemic health, including enhancements in cardiometabolic risk, systemic inflammation, and the occurrence of preterm deliveries. Outcomes on the quality of life, however, were scarce and should be considered in further studies.

Regarding the OHRQoL, a randomized clinical trial^172^ compared the immediate postoperative quality of life of patients who have undergone different treatment approaches. Patients were treated with nonsurgical therapy, surgical or surgical plus enamel matrix protein derivative (EMD). Quality of life was assessed after 1 week using OHIP‐14 and GOHAI. The findings of the study revealed that whereas no differences were observed at baseline, at 1 week, individuals treated by surgery reported worse OHRQoL. Therefore, patient perceptions were better for the nonsurgical or surgical plus EMD approaches. These findings were corroborated by an umbrella review that stressed that the impact of surgical therapy on OHRQoL may not be as significant as nonsurgical therapy.^121^ On the other hand, the results obtained with nonsurgical therapy do not seem to be influenced by instruments (e.g., lasers) or a number of interventions (quadrant vs. full‐mouth disinfection).^170^

Peikert et al.^167^ investigated the improvements of nonsurgical periodontal therapy on OHRQoL in a cohort of 172 patients. The authors observed that the OHIP‐14 scores improved significantly 6–8 weeks after treatment in patients with moderate and severe periodontitis. The caregiver and treatment modality also seemed to influence the scores. The use of air‐powder devices and adjunct systemic antibiotics significantly improved OHIP scores in comparison to other treatment alternatives such as laser, photodynamic therapy, curettes alone or in combination with ultrasonic scaling device, and chlorhexidine. Therefore, it can be suggested that the different approaches to the treatment of periodontitis can improve a patient's quality of life.

#### 5.1.3 Supportive periodontal therapy

Adherence to a regular supportive care program has been suggested as a key factor in the long‐term maintenance of the treatment outcomes.^88^, ^173^ El Sayed et al.^174^ assessed the OHRQoL 20 years after periodontal treatment in patients diagnosed with chronic periodontitis. Of 63 patients examined, up to 75% showed no signs of disease recurrence, inflammation, or masticatory dysfunction. Overall, around 50% of the patients reported an OHIP‐49 score of ≤10, indicating still some impairment, and 11% reported no impairment at all. In addition, smokers reported less‐favorable OHRQoL than nonsmokers, as well as compliant patients versus noncompliers and patients with a severe form of periodontitis versus less‐severe forms. A subgroup analysis showed that patients with severe chronic periodontitis who were compliant with SPC had lower OHIP scores (median 7) than erratic (median 12) and noncompliant patients (median 14.5). These findings support the assumption that adherence to supportive care is important for the maintenance of oral health. A long‐term retrospective study^175^ compared tooth loss in 51 periodontally healthy/gingivitis and 56 periodontally compromised patients during 15–25 years of follow‐up. Both groups exhibited similar tooth loss rates (39/38 patients/group, respectively), albeit only the periodontally compromised group lost teeth due to periodontitis (11 patients). Comparable satisfaction regarding aesthetics, chewing function, hygienic ability, and OHIP score were observed between the groups.

On the other hand, patients without regular supportive care tend to present poorer OHRQoL. A retrospective cohort study^176^ assessed the OHRQoL outcomes of young adults with advanced periodontitis. After 7–26 years of periodontal treatment, patients experienced disease recurrence and tooth loss. Smoking was associated with progression of the disease. Substantial tooth loss and higher residual pockets led to higher OHIP scores, that is, poorer OHRQoL outcomes. A systematic review^136^ reported similar findings. Hence, it can be suggested that the secondary prevention of periodontitis (supportive care), including management of risk factors such as smoking, can maintain the quality‐of‐life scores achieved after active periodontal therapy in the long term.^177^

#### 5.1.4 Management of sequelae

Stage IV periodontitis is the most severe form of periodontitis, with loss of a minimum of five teeth and a need for complex rehabilitation due to masticatory dysfunction.^1^, ^88^ It significantly reduces the quality of life of patients.^136^ Numerous treatment approaches can be used to rehabilitate patients who lost teeth, associating implants or not.^178^, ^179^, ^180^ Gennai et al.^181^ investigated, in a systematic review, the effect of rehabilitation in fully/partially edentulous patients diagnosed with Stage IV periodontitis on OHRQoL and systemic health. Of 59 articles included, some affected by periodontitis and others not specified, OHRQoL improved throughout all the studies, irrespective of the number of missing teeth, location, or treatment modality. At the same time, nonrehabilitated subjects showed inferior cognitive status, higher medication intake, and frailty.

In summary, periodontitis can be asymptomatic in the early stages, but soon it becomes symptomatic. There is a wide body of evidence on the association between periodontitis and impaired oral health‐related quality of life across a range of different populations and settings. It can compromise masticatory function, speech, food intake, comfort, appearance, and self‐confidence. After treatment interventions, the OHRQoL is improved and, under regular supportive care and control of risk factors, can be maintained in the long term. Tooth loss has a strong impact on OHRQoL. The rehabilitation of these sites is important to stabilize these cases and to restore patients' quality of life.

### 5.2 Impact of peri‐implantitis on the quality of life

#### 5.2.1 The diagnosis of peri‐implantitis

The awareness of having peri‐implantitis may be an unpleasant surprise for the patient. Symptoms take more time to appear, and some individuals are not aware that such complications could happen.^182^ There are few studies on the impact of peri‐implantitis on OHRQoL. A qualitative study^183^ assessed 15 patients' reactions after being diagnosed with peri‐implantitis, their opinions on dental implant therapy, and expectations of the treatment of the disease. The interviews revealed that individuals had very high expectations in the beginning, considering dental implant therapy as a permanent solution to oral/dental problems. The referral and diagnosis of peri‐implantitis, however, was seen as a stressful event with consequences on mental well‐being and daily life. Individuals were not only worried about losing their implants or the effects of peri‐implant infection but also about the financial costs that would follow treatment of the disease.

Insua et al.^184^ assessed the impact of different aspects on patients' quality of life (QoL) of individuals undergoing implant maintenance care. Information regarding the level of knowledge, awareness, and attitudes about peri‐implantitis, orientations provided by dentists/specialists who performed the treatment and its perceptions, and the level of satisfaction were collected using a questionnaire. The quality of life was assessed by 15 items in a Likert scale format as part of the Michigan Oral Health QoL Scale. As a result, of 135 patients included, 17.8% were diagnosed with peri‐implantitis. Most patients (74.1%), however, did not know what peri‐implantitis was. Few patients (8%) reported that the disease impaired their life in all aspects. Some individuals (12.5%) also reported reduced general happiness due to their implant. Besides, 32% of patients reported that the disease limited the kind and types of food that they could eat, and 8% described a bad taste of food. An additional 48% of individuals suffered discomfort due to the disease. Therefore, QoL was impaired by the presence of peri‐implantitis, with a high level of concern and a low level of therapeutic satisfaction.

Recently, Esteve‐Pardo et al.^185^ assessed the impact of implant‐related complications on different aspects in a cross‐sectional study. After the clinical exam, the clinician gave a description of the complication and its general prognosis according to the literature without giving a specific treatment plan and its cost. Then, patients were asked to complete a form. Using five visual numerical scales, ranging from 0 to 10 and three endpoint descriptors, the following five items were scored by patients: perceived pain, functional impairment, concern, quality of life (QoL), and confidence of the patients with potential new dental implant treatments. Three types of complications were considered biological, when signs of peri‐implant disease were present; mechanical, when technical problems were included; and mixed, when a combination of both was found. As a result, 408 patients were included, of which 26% had biological complications, and 4.4% had a mixture of both technical and biological. About 1 of 3 patients (28.9%) presented signs of peri‐implant disease. In the presence of biological/mixed complications, pain was higher, chewing impairment was correlated with implant loss or removable/full‐arch prosthesis, patients were concerned and scored more negatively if the diseased implant was in the esthetic area, or if the implant was placed in another clinic. In addition, women and patients with biological complications were found to be less confident with new treatments than the average. Finally, the study showed that complications have caused the patient, to a moderate degree, concerns, functional impairment, pain, and decreased QoL. Therefore, the impact of implant‐related complications on patients' perceptions should be neglected.

On the other hand, Romandini et al.^186^ assessed the symptoms and perception of peri‐implant diseases reported by patients, and their signs and potential impact on the oral health quality of life, in a cross‐sectional study. In most cases, individuals were blinded about their peri‐implant status (except for patients with previous diagnoses). They were assessed in two steps: a self‐reported questionnaire that included the OHIP‐14 and a structured interview. The structured interview was performed by a trained interviewer with standardized questions. Patients were asked to answer their perception concerning the peri‐implant health status and any history of pain, spontaneous discomfort, bleeding, suppuration, swelling, and discomfort during brushing for each implant, with the aid of a facial mirror to precisely localize it. Among 99 patients and 458 implants included, the prevalence of peri‐implantitis was 56.6% and 28%, at patient and implant levels, respectively. Nevertheless, 91.7% of the implants were perceived as healthy by the patients. Even in the case of peri‐implantitis, 88.9% of the implants were perceived as healthy. The OHIP‐14 indicated a low level of OHRQoL impairment. Hence, although symptoms and patient's perception of periodontitis have been widely studied in the literature and may include gingival bleeding during toothbrushing and tooth mobility,^128^ the symptoms and patient perception of peri‐implant diseases and also their impact on OHRQoL were negligible. On the opposite side to what is known for periodontitis, the impact of peri‐implant diseases on patients' OHRQoL could only be perceived after the loss of osseointegration (implant loss). These findings highlighted the importance of regular SPC, not only as a preventive approach but also to allow for early diagnosis/intervention in patients with dental implants considering that peri‐implant diseases are frequently asymptomatic.

#### 5.2.2 Treatment of peri‐implantitis

The efficacy of various interventions to treat peri‐implantitis is still a matter of debate due to its poor predictability in the long term.^7^, ^40^, ^41^, ^187^, ^188^, ^189^ Nevertheless, the few studies that reported patient‐related outcomes have shown that peri‐implant diseases are silent in most cases. Following the surgical treatment, however, some pain or discomfort may be experienced. If the implant surface gets exposed by the recession of the mucosa, more space between the soft tissues and supra‐structure might negatively affect aesthetics and phonetics.^190^ A recent prospective study^190^ described the long‐term patient‐reported outcomes following surgical treatment of peri‐implantitis. Forty‐three patients with 116 implants treated for peri‐implantitis by resective surgery completed the OHIP‐14 questionnaire 1 week before and 6, 18, and 36 months after the procedure. As a result, the mean total score of OHIP‐14 was low at 1‐week presurgery, and 6, 18, and 36 months postsurgery, 7.2 (SD 7.3), 6.0 (SD 6.9), 6.8 (SD 9.7), and 7.0 (SD 9.4), respectively. No significant differences were observed over time in relation to the seven domains. In addition, 70% of the total scores in the OHIP‐14 questionnaire were “0”, that is, these patients reported that they “never” experienced problems related to an OHRQoL question. It can be suggested that the quality of life of most patients was not influenced by peri‐implantitis or the treatment performed. Interestingly, the authors reported that the domain “psychological discomfort” had the highest scores, demonstrating that the discomfort of having peri‐implantitis may be more critical than the other aspects. One aspect very important to highlight is that the study was conducted in a university setting, and it might have affected the patients psychologically since they did not have to pay any fee for the surgical treatment or the follow‐up. Other treatment approaches, especially regenerative interventions, may have a different impact on the patient's quality of life and must be further investigated.

Recently, a qualitative interview study^191^ assessed peri‐implantitis patients' sensations, expectations, and experiences throughout the entire process of dental implants, the disease, as well as undergoing treatment with laser or mucosal flap surgery. Eighteen individuals were treated either with open‐flap debridement, including cleaning of the implant surface with the aid of a brush, or semi‐surgical laser treatment (970 nm laser). They were interviewed about 14 days after treatment. Questions were designed and phrased around three topics: living with dental implants (comparison of teeth and implants, placing the dental implants, oral hygiene habits, and expected survival of the implants), diagnosis of peri‐implantitis and included symptoms, and experiences of the treatment of peri‐implantitis. The first topic, losing teeth had a negative implication; patients reported that it was psychologically tough, associated with sadness, loss of functional chewing ability, and high cost for rehabilitation. They were receiving dental implants felt like a positive occurrence but a costly, mechanical, and extensive journey. After implant placement, some patients described it as a part of them, and others reported a different feel. The diagnosis of peri‐implantitis impacted the individuals' quality of life in different aspects. Regardless of whether they were symptomatic or not, their quality of life was negatively impacted after the diagnosis. It was a surprise or shock to some participants. The symptoms reported were pulsating and pounding, soreness, light swelling, bleeding during brushing, and a feeling of inflammation. Individuals were worried, feared, and frustrated with the diagnosis. There was a clear lack of knowledge of the existence of the disease. Regarding the treatment process, individuals are expected to become disease free, keep their implants, and avoid recession of the mucosa. Those who were treated by open‐flap surgery were nervous and thought it could hurt. The laser patients were excited about the novel treatment. In general, this qualitative study showed that patients perceived losing a tooth as very frustrating, with a negative impact on their lives. The onset and diagnosis of peri‐implantitis were not noticed by some individuals, but all felt a sense of nervousness before the treatment. The treatment of peri‐implantitis leads to, in general, only slight discomfort.

#### 5.2.3 Management of sequelae

Implant removal is often the treatment of choice to effectively arrest peri‐implantitis.^192^ Cases in which the bone loss exceeds 50% of the total length of the infected implant or cases of disease recurrence are frequently indicated for implant removal due to its unfavorable prognosis.^193^, ^194^, ^195^, ^196^, ^197^ A cross‐sectional study^198^ evaluated the sequelae and patient satisfaction after implant removal. Clinical parameters, including symptomatology, were assessed before and after implant removal. Patients were asked to fill in a questionnaire after implant removal, using a Likert scale, including their satisfaction with the treatment they received; if they received a new implant, how satisfied they were, satisfaction with the previous surgical and prosthetic part of the treatment; and if they would place more implants in the future. In addition, the Oral Health Impact Profile‐14 (OHIP‐14) was completed. Patient's expectations and their willingness to receive a new implant were also determined. Of 31 patients with 45 implants analyzed, 64.5% were removed due to peri‐implantitis. Signs of infection (51.7%), bleeding on probing (37.5%), foreign body sensation (25%), mobility (20.7%), and pain (19.4%) were often observed. After implant removal, few patients experienced pain (10%), bleeding (7.7%), or signs of infection (3.4%). Guided bone regeneration was a common intervention applied simultaneously to implant removal (74.1%). Overall, the reported patients' satisfaction was high. The presence of signs of infection significantly influenced the level of patient satisfaction and the final OHIP‐14 score. The median OHIP‐14 score was 8.2 when infection or suppuration was present and 3.3 when it was absent. In addition, patients' attitudes changed in relation to placing new implants at the same clinic or with the same professional as before and regarding maintenance appointments, adhering to a strict protocol.

As with periodontitis, peri‐implantitis is, in most situations, asymptomatic and not perceived by the patients. Studies that reported an impaired quality of life reported it for a moderate degree only, and the examiners had given some description of their peri‐implant diagnosis prior to the questionnaire. Nevertheless, the evidence is still scarce. Therefore, more studies are necessary to elucidate the impact of peri‐implantitis on the patient's quality of life.

## AUTHOR CONTRIBUTIONS

Muhammad H. A. Saleh conceived the ideas and drafted the manuscript. Debora R. Dias collected and analyzed the data and drafted the manuscript. Purnima Kumar led the writing and provided a critical review of the manuscript.

## CONFLICT OF INTEREST STATEMENT

Apart from the support of the authors' institution, no external funding was available for this study. The authors declare that there are no conflicts of interest in this study.

## Data Availability

The data supporting this study's findings are available from the corresponding author upon reasonable request.
